# FAM111A is dispensable for electrolyte homeostasis in mice

**DOI:** 10.1038/s41598-022-14054-8

**Published:** 2022-06-17

**Authors:** Barnabas P. Ilenwabor, Heidi Schigt, Andreas Kompatscher, Caro Bos, Malou Zuidscherwoude, Bram C. J. van der Eerden, Joost G. J. Hoenderop, Jeroen H. F. de Baaij

**Affiliations:** 1 Department of Physiology, Radboud Institute for Molecular Life Sciences, Radboud University Medical Center, P.O. Box 9101, 6500 HB Nijmegen, The Netherlands; 2 Department of Internal Medicine, Erasmus MC, Erasmus University Medical Center, Rotterdam, The Netherlands

**Keywords:** Calcium and vitamin D, Kidney

## Abstract

Autosomal dominant mutations in *FAM111A* are causative for Kenny-Caffey syndrome type 2. Patients with Kenny-Caffey syndrome suffer from severe growth retardation, skeletal dysplasia, hypoparathyroidism, hypocalcaemia, hyperphosphataemia and hypomagnesaemia. While recent studies have reported FAM111A to function in antiviral response and DNA replication, its role in regulating electrolyte homeostasis remains unknown. In this study, we assessed the role of FAM111A in the regulation of serum electrolyte balance using a *Fam111a* knockout (*Fam111a*^−*/*−^) C57BL/6 N mouse model. *Fam111a*^−*/*−^ mice displayed normal weight and serum parathyroid hormone (PTH) concentration and exhibited unaltered magnesium, calcium and phosphate levels in serum and 24-hour urine. Expression of calciotropic (including *Cabp28k, Trpv5, Klotho* and *Cyp24a1*), magnesiotropic (including *Trpm6*, *Trpm7*, *Cnnm2* and *Cnnm4*) and phosphotropic (*Slc20a1*, *Slc20a2*, *Slc34a1* and *Slc34a3*) genes in the kidneys, duodenum and colon were not affected by *Fam111a* depletion. Only *Slc34a2* expression was significantly upregulated in the duodenum, but not in the colon. Analysis of femurs showed unaffected bone morphology and density in *Fam111a*^*−/−*^ mice. Kidney and parathyroid histology were also normal in *Fam111a*^*−/−*^ mice. In conclusion, our study is the first to characterise the function of FAM111A in vivo and we report that mice lacking FAM111A exhibit normal electrolyte homeostasis on a standard diet.

## Introduction

Kenny-Caffey syndrome type 2 (KCS2, OMIM #127000) is a rare hereditary disorder characterised by short stature, cortical thickening and medullary stenosis of the long tubular bones, delayed closure of the anterior fontanelle, hypoparathyroidism and electrolyte imbalances: hypocalcaemia, hyperphosphataemia and hypomagnesaemia^1–4^. In addition, dental and ocular defects are frequently observed^5,6^. While KCS2 patients can reach adulthood, a similar but more severe disorder called gracile bone dysplasia (GCLEB, OMIM #602361) is perinatally lethal^7^. Both KCS2 and GCLEB were found to be caused by mutations in the *FAM111A* gene located on chromosome 11q12.1^3,4^. While all known mutations are located in and around the serine peptidase domain and the autocleavage site, the specific mutations are different between KCS2 and GCLEB^3,4,6–17^.

Over the last years, several studies aimed to investigate the function of FAM111A in more detail. FAM111A has been postulated to be a protease expressed in a cell-cycle dependent manner that exhibits autocleavage^18,19^. It plays a role in DNA replication, loading proliferating cell nuclear antigen (PCNA) onto chromatin^19^ and preventing fork stalling, potentially through proteolytic removal of protein obstacles from the genomic DNA^18^. While FAM111A is required for DNA replication^19^, its overexpression also reduces DNA replication^19,20^ and leads to caspase-dependent programmed cell death in the osteosarcoma cell line U2OS^20^. In addition, FAM111A was identified as a host range restriction factor, meaning that it could prevent viral replication by certain viruses, in particular simian vacuolating virus 40 (SV40), rabbitpox virus and vaccinia virus^21,22^. This effect is achieved through inhibition of viral replication centre formation and viral DNA replication by disrupting the host cell nuclear barrier function^23,24^. Viruses that contain SV40 large T antigen (LT) or serine protease inhibitor 1 (SPI-1), on the other hand, can overcome the host defence through inhibition of FAM111A^21,22^. While we are beginning to understand some of the molecular functions of FAM111A, it remains unclear how these relate to the observed phenotype in KCS2 and GCLEB patients.

Regulation of Ca^2+^ and Mg^2+^ homeostasis mainly occurs by absorption from the intestine, storage in bone and reabsorption by the kidneys. The thick ascending limb (TAL) and the distal convoluted tubule (DCT) segments of the nephron are important for the renal handling of these ions by expressing specific channels and transporters, thereby regulating ion excretion into the urine according to the requirements of the body^25,26^. Disturbed electrolyte homeostasis is an essential feature of KCS2, but the primary cause of the electrolyte disturbances has not been established. Hypoparathyroidism has been proposed as the main cause, as parathyroid tissue was absent in a KCS2 patient^27^. Indeed, primary hypoparathyroidism is in line with patients’ increased bone density as well as hypocalcaemia and hypomagnesaemia. However, Isojima and coworkers demonstrated that magnesium supplementation restored parathyroid and calcium levels in a patient, suggesting that hypomagnesaemia is the primary defect^3^. Interestingly, in a disorder called hypomagnesaemia with secondary hypocalcaemia (HSH), caused by mutations in the magnesium channel TRPM6, patients also present with secondary hypoparathyroidism^28^. Of note: hypoparathyroidism only occurs as a consequence of hypomagnesaemia if serum magnesium levels are severely low^29^. A major obstacle to the investigation of FAM111A in electrolyte homeostasis is the absence of a suitable physiological study model, as cell models do not represent the complex interplay between organs or cell types in vivo.

To increase our understanding of the electrolyte disturbances in KCS2 patients, we generated *Fam111a* knockout (*Fam111a*^*−/−*^), heterozygous (*Fam111a*^+*/−*^) and wild type (*Fam111a*^+*/*+^) mice. The mice were kept in metabolic cages to collect faeces, blood and urine for biochemical analysis. The parathyroid glands, kidneys and intestines were subjected to immunohistochemistry and expression analyses, and bone structure was analysed by micro-computed tomography (micro-CT).

## Results

### Mouse breeding

**Figure 1:**
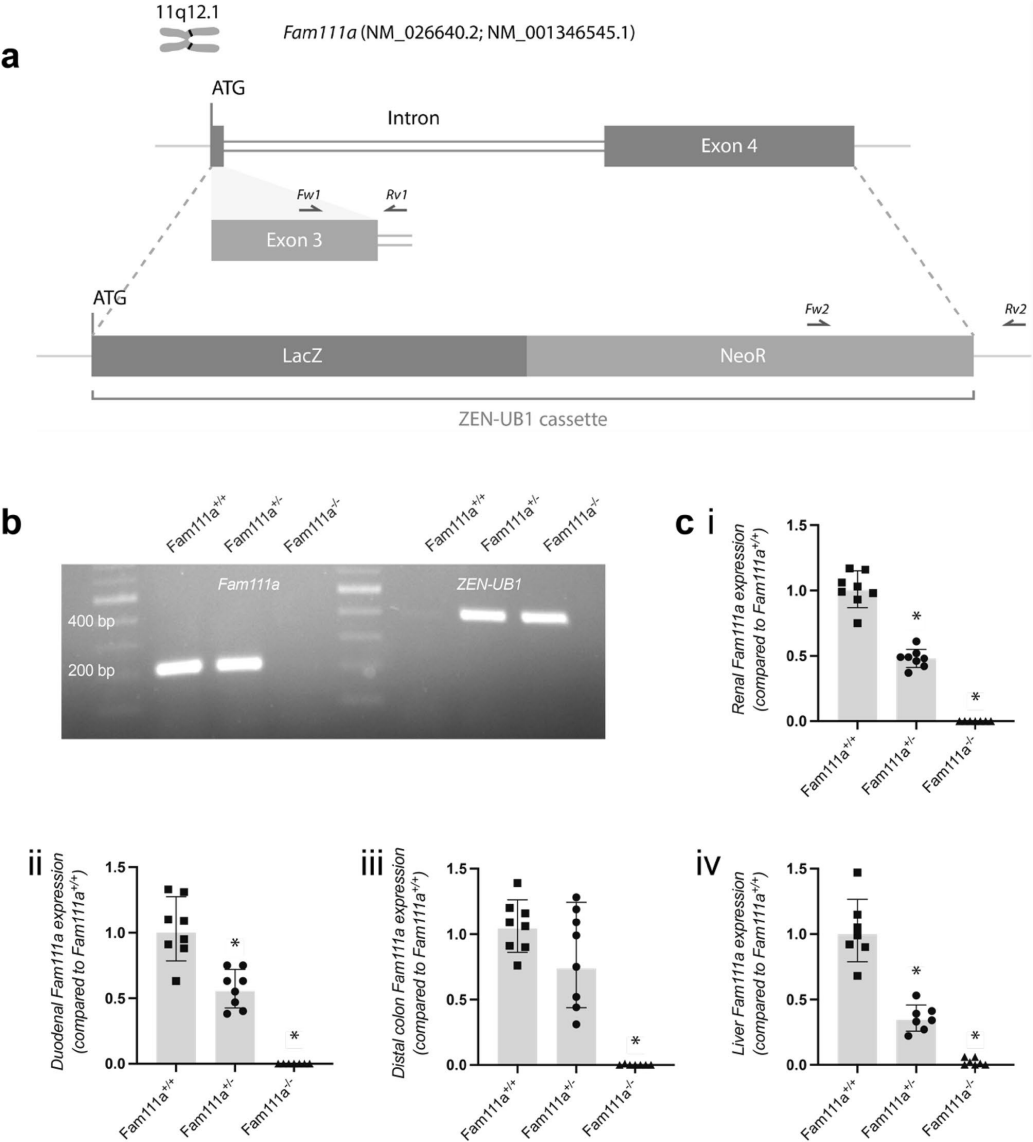
Mouse breeding strategy and genotyping. (**A**) The ZEN-UB1 cassette was inserted to replace the protein coding region of *Fam111a* in ES cells. Forward (Fw) and reverse (Rv) primers used for genotyping the mice are represented with arrows. Primer pair 1 recognises wildtype *Fam111a* and primer pair 2 recognises the ZEN-UB1 cassette. (**B**) PCR products showing the genotype of *Fam111a*^+*/*+^, *Fam111a*^+*/*−^ and *Fam111a*^−*/*−^ mice at the expected sizes. The full-length gel is presented in Supplementary Figure S1. (**C**) mRNA expression of *Fam111a* (i) in the kidney, (ii) proximal duodenum, (iii) distal colon and (iv) liver tissues confirming the genotype of *Fam111a*^+*/*+^, *Fam111a*^+*/*−^ and *Fam111a*^−*/*−^ mice 8–10 weeks old. n = 8 (4 males, 4 females) for *Fam111a*^+*/*+^ and *Fam111a*^+*/−*^, n = 7 (3 males, 4 females) for *Fam111a*^−*/*−^ mice. For liver, n = 7 (3 males, 4 females) for *Fam111a*^+*/*+^ and n = 7 (4 males, 3 females) for *Fam111a*^+*/*−^ and n = 7 (3 males, 4 females) for *Fam111a*^−*/*−^ mice. Results were normalised to *Gapdh* expression (reference gene). Data are presented as mean ± standard deviation (SD). Significance was determined using one-way ANOVA followed by Tukey’s post-hoc test. *p < 0.05 compared to *Fam111a*^+/+^ group.

To generate *Fam111a*^+*/−*^ mice, the protein coding region of *Fam111a* was substituted by the ZEN-Ub1 cassette which contains the *E. coli* *lacZ* reporter gene and neomycin resistance gene (Fig. 1A). Breeding *Fam111a*^+*/−*^ mice resulted in the birth of 43 pups in the ratio *Fam111a*^+*/*+^ (15), *Fam111a*^+*/−*^ (20) and *Fam111a*^*−/−*^ (8), which was not significantly different from the expected proportion (χ^2^ test, *p* > 0.2). The male to female ratios of the animals used for the study were 4:4, 5:3 and 3:4, respectively. Genotyping confirmed the presence of the cassette in *Fam111a*^*−/−*^ and *Fam111a*^+*/−*^ mice and the *Fam111a* allele in *Fam111a*^+*/*+^ and *Fam111a*^+*/−*^ animals (Fig. 1B). Deletion of *Fam111a* was also confirmed at the RNA level by real-time quantitative polymerase chain reaction (RT-qPCR) using RNA isolated from the kidneys, liver, proximal duodenum and distal colon. *Fam111a* expression was significantly reduced (*p* < 0.05) by approximately 50% in all tested tissues except the distal part of the colon of *Fam111a*^+*/−*^ mice compared to the *Fam111a*^+*/*+^ group. *Fam111a* expression was absent in the *Fam111a*^*−/−*^ mice in all the measured tissues (Fig. 1C).

### Fam111a^−/−^ mice do not exhibit growth and electrolyte disturbances

To investigate the effect of *Fam111a* deletion on the weight and electrolyte levels in mice, *Fam111a*^+*/*+^*, Fam111a*^+*/−*^* and Fam111a*^*−/−*^ mice were placed in metabolic cages. *Fam111a* deletion did not affect the weight of the mice significantly compared to their heterozygotes and wild type littermates. There were also no significant differences in food and water consumption, urine volume and faecal weight between the groups (Table 1).

**Table 1:** Metabolic cage parameters.

	*Fam111a*^+*/*+^	*Fam111a*^+*/*−^	*Fam111a*^−*/*−^
Weight (g)	23.9 ± 3.9	25.1 ± 4.3	20.6 ± 2.1
Food intake (g)	4.4 ± 0.7	4.9 ± 0.5	4.1 ± 0.5
Water intake (ml)	5.3 ± 1.7	5.1 ± 1.3	4.1 ± 0.5
Faecal weight (g)	2.2 ± 0.7	2.4 ± 0.5	1.9 ± 0.5
Urine volume (ml)	0.8 ± 0.3	0.9 ± 0.4	0.8 ± 0.3

Average values of metabolic cage parameters ± SD of *Fam111a*^+*/*+^ (4 males, 4 females)*, Fam111a*^+*/*−^ (4 males, 4 females) *and Fam111a*^−*/*−^ (3 males, 4 females) mice, 8–10 weeks old.

Calcium (Ca^2+^) levels, which are decreased in the serum of KCS2 patients, did not differ between *Fam111a*^+*/*+^*, Fam111a*^+*/−*^* and Fam111a*^*−/−*^ mice in serum (1.98 ± 0.04 vs 2.06 ± 0.03 vs 2.04 ± 0.04 mmol/L, respectively; *p* > 0.2) and in urine (2.0 ± 0.2 vs 2.2 ± 0.3 vs 2.0 ± 0.2 μmol/24 h, respectively; *p* > 0.2) (Table 2). Serum magnesium (Mg^2+^) concentration is low in KCS2 patients, but was not altered in the *Fam111a*^*−/−*^ mice compared to *Fam111a*^+*/*+^ and *Fam111a*^+*/−*^ groups (1.21 ± 0.03 vs 1.08 ± 0.05 and 1.09 ± 0.03 mmol/L, respectively; *p* = 0.062 using one way ANOVA). Urine excretion of Mg^2+^ was similar in *Fam111a*^+*/*+^*, Fam111a*^+*/−*^ and *Fam111a*^*−/−*^ mice (23.2 ± 3.0, 34.8 ± 4.6 and 35.1 ± 4.4 μmol/24 h, respectively; *p* = 0.08 using one way ANOVA). Serum phosphate (PO_4_^3−^) levels are high in KCS2 patients, but no differences were found between the *Fam111a*^+*/*+^*, Fam111a*^+*/−*^ and *Fam111a*^*−/−*^ mice (2.2 ± 0.1, 2.1 ± 0.1 and 1.9 ± 0.1 mmol/L, respectively; *p* > 0.2) and urine PO_4_^3−^ levels were unchanged (52 ± 7, 62 ± 10 and 64 ± 9 μmol/24 h, respectively; *p* > 0.2). There were no differences in serum sodium (Na^+^) levels between the groups (152 ± 1 vs 153 ± 1 vs 151 ± 1 mmol/L, respectively; *p* > 0.2) and urine Na^+^ was similar for all the groups as well (170 ± 21 vs 174 ± 25 vs 167 ± 23 μmol/24 h, respectively; *p* > 0.2). Finally, potassium (K^+^) levels were not altered in serum (5.4 ± 0.3 vs 5.3 ± 0.1 vs 5.8 ± 0.2 mmol/L, respectively; *p* > 0.2) and urine (571 ± 41 vs 632 ± 73 vs 585 ± 55 μmol/24 h, respectively; *p* > 0.2) of *Fam111a*^+*/*+^*, Fam111a*^+*/−*^ and *Fam111a*^*−/−*^ mice. Albumin serum levels were measured as a control and were the same in each group (4.10 * 10^–3^ ± 0.4 * 10^–3^ vs 4.13 * 10^–3^ ± 0.4 * 10^–3^ vs 4.16 * 10^–3^ ± 0.4 * 10^–3^ mmol/L; *p* > 0.2).

**Table 2:** Serum and urine electrolyte values.

	Serum (mmol/L)			Urine (μmol/24 h)		
	*Fam111a*^+*/*+^	*Fam111a*^+*/*−^	*Fam111a*^−*/*−^	*Fam111a*^+*/*+^	*Fam111a*^+*/*−^	*Fam111a*^−*/*−^
Mg^2+^	1.08 ± 0.15	1.09 ± 0.08	1.21 ± 0.09	23.2 ± 8.3	34.8 ± 13.0	35.1 ± 11.5
Ca^2+^	1.98 ± 0.11	2.06 ± 0.09	2.04 ± 0.09	2.0 ± 0.5	2.2 ± 0.8	2.0 ± 0.6
PO_4_^3−^	2.2 ± 0.3	2.1 ± 0.3	1.9 ± 0.3	52 ± 20	62 ± 28	64 ± 24
Na^+^	152 ± 2	153 ± 2	151 ± 1	170 ± 58	174 ± 67	167 ± 61
K^+^	5.4 ± 0.8	5.3 ± 0.3	5.5 ± 0.5	571 ± 115	632 ± 193	585 ± 146
Albumin	4.1 * 10^–3^ ± 0.4 * 10^–3^	4.1 * 10^–3^ ± 0.4 * 10^–3^	4.2 * 10^–3^ ± 0.4 * 10^–3^	–	–	–

Average serum and urine electrolyte values ± SD measured in *Fam111a*^+*/*+^ (4 males, 4 females)*, Fam111a*^+*/*−^ (4 males, 4 females) mice, 8–10 weeks old.

### Expression of transporter genes is unchanged in the kidneys of Fam111a^−/−^ mice

**Figure 2:**
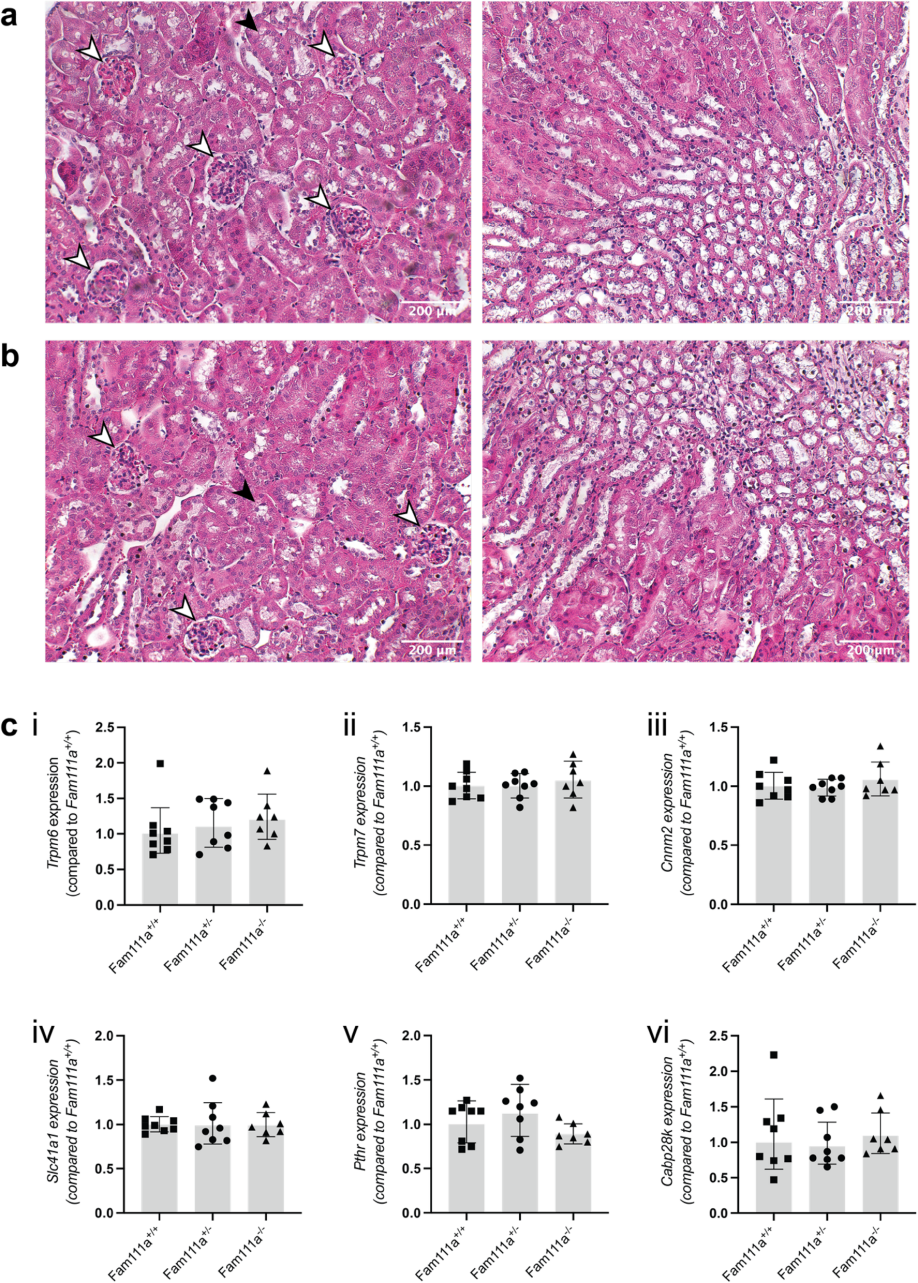

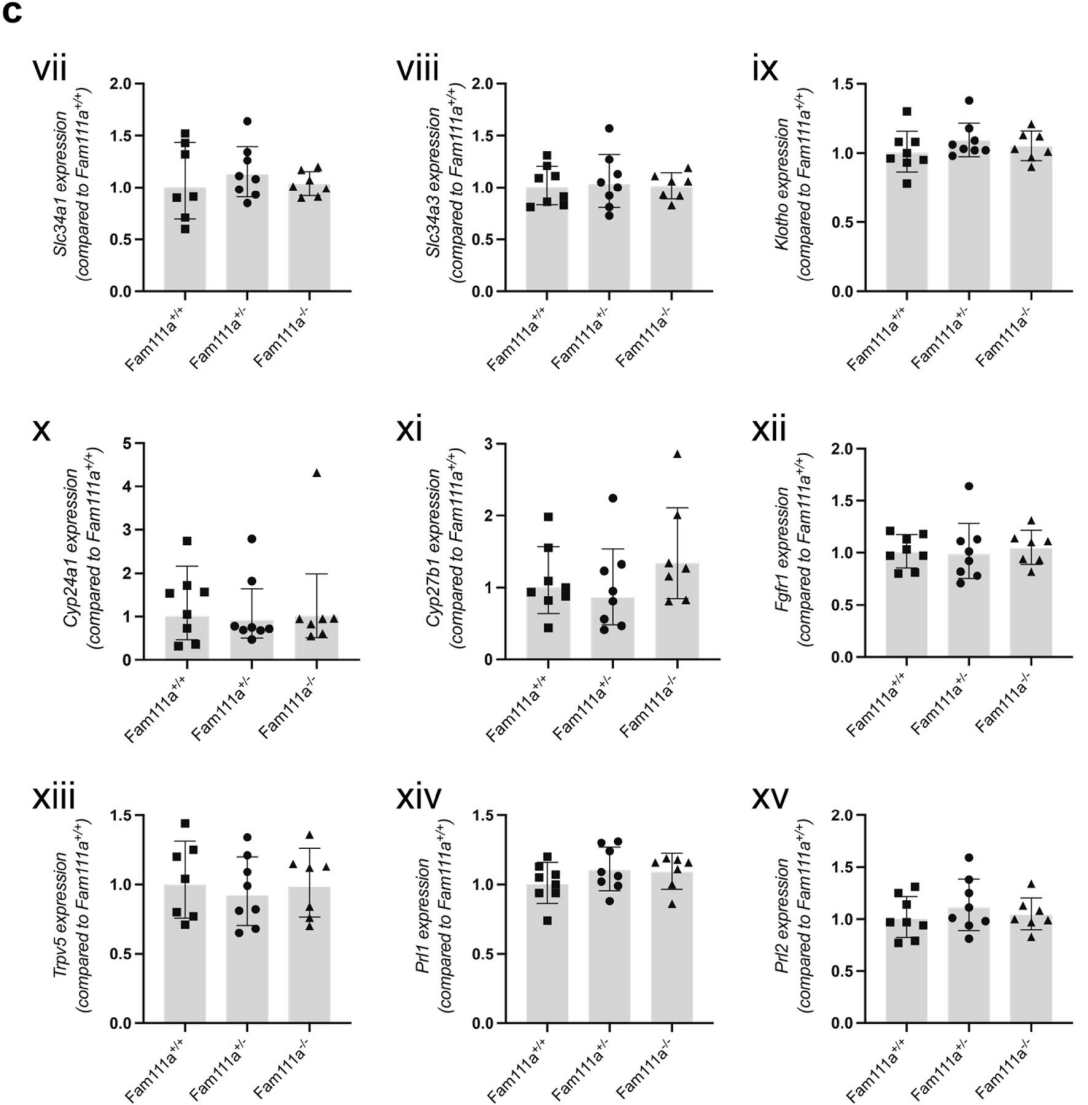
Kidney histology and gene expression of renal electrolyte transporters in *Fam111a*^−*/*−^ mice. (**A**) Representative H&E staining of kidney tissues from *Fam111a*^+*/*+^ and (**B**) *Fam111a*^−*/*−^ mice showing cortex (left) and medulla (right). Glomerular (white arrows) and tubular structures (black arrows) are indicated. (**C**) mRNA expression levels of (i) *Trpm6*, (ii) *Trpm7*, (iii) *Cnnm2*, (iv) *Slc41a1*, (v) *Pthr*, (vi) *Cabp28k*, (vii) *Slc34a1*, (viii) *Slc34a3*, (ix) *Klotho*, (x) *Cyp24a1*, (xi) *Cyp27b1*, (xii) *Fgfr1*, (xiii), *Trpv5*, (xiv) *Prl1* and (xv) *Prl2* in the kidneys of *Fam111a*^+*/*+^*, Fam111a*^+*/*−^ and *Fam111a*^−*/*−^ mice are similar. n = 8 (4 males, 4 females) for *Fam111a*^+*/*+^ and *Fam111a*^+*/*−^, n = 7 (3 males, 4 females) for *Fam111a*^−*/*−^ mice. n = 7 (3 males, 4 females) for *Fam111a*^+*/*+^ mice for the *Trpv5* and *Slc34a1* genes. Results were normalised to *Gapdh* expression (reference gene). Data are presented as mean ± SD. Significance was determined using one-way ANOVA.

The kidneys play an important role in the regulation of serum electrolyte levels. Haematoxilin and eosin (H&E) staining of the kidney tissue showed normal glomerular and tubular structures (white and black arrowheads, respectively) in *Fam111a*^*−/−*^ animals and no fibrosis or infiltration of immune cells was observed (Fig. 2A,B). To identify potential renal compensatory mechanisms, expression of genes involved in Ca^2+^ and Mg^2+^ handling was measured in the kidneys. Expression of the magnesiotropic genes including *Trpm6*, *Trpm7*, *Cnnm2*, *Slc41a1, Prl1* and *Prl2* was not different between the groups (Fig. 2C). The expression of renal Ca^2+^ transport genes *Trpv5* and *Cabp28k* was stable upon *Fam111a* deletion in mice compared to the wild type animals. There were also no significant differences in the expression of renal sodium/phosphate cotransporter genes *Slc34a1* and *Slc34a3* (Fig. 2C) as well as in *Cyp27b1*, *Cyp24a1* and *Pthr1*, which are involved in vitamin D metabolism and PTH signaling (Fig. 2C).

### Transcriptional profile of kidneys from Fam111a^+/+^ and Fam111a^−/−^ mice using RNA sequencing

RNA sequencing (RNAseq) was performed to identify differences in the RNA transcriptional profile of kidneys from *Fam111a*^*−/−*^ compared with *Fam111a*^+*/*+^ mice. The expression of *Fam111a* and the mitochondrial genes Glycine N-acyltransferase (*Glyat)* and Glycine N-acyltransferase-like protein (*Gm4952)* was significantly downregulated in the *Fam111a*^*−/−*^ mice compared to the *Fam111a*^+*/*+^ group (*p* < 0.05; FDR < 0.05). Solute carrier protein 47 member 1 (*Slc47a1*) and the E3 ubiquitin-protein ligase Midline-1 (*Mid1)* showed significantly higher level of transcripts in *Fam111a*^*−/−*^ mice than in the *Fam111a*^+*/*+^ group (*p* < 0.05; FDR < 0.05).

### Fam111a^−/−^ mice possess a similar distal convoluted tubule content compared to Fam111a^+/+^ mice

**Figure 3:**
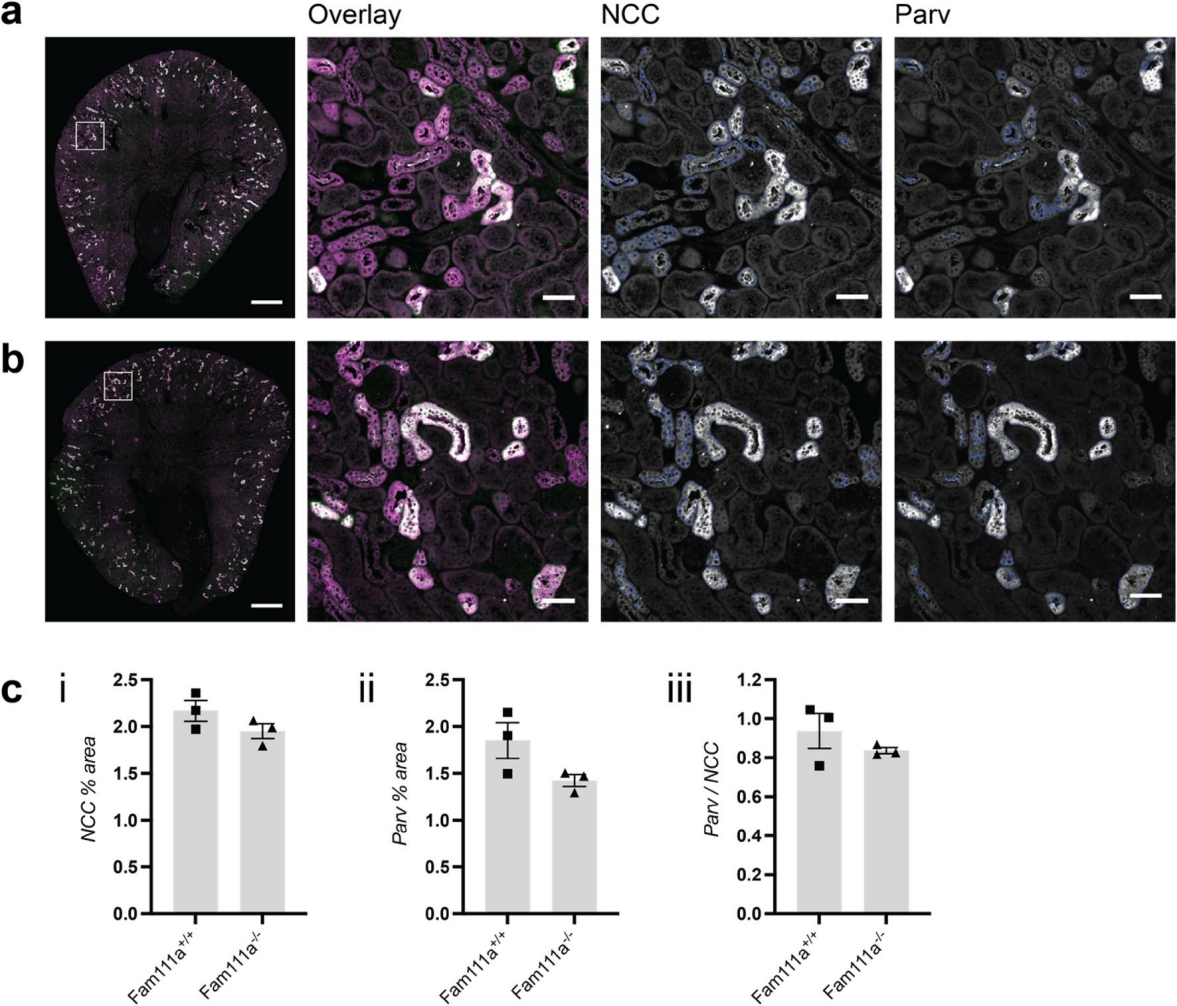
DCT area is unaltered in *Fam111a*^−*/*−^ mice compared to *Fam111a*^+*/*+^ mice. Representative images of whole kidney slices (left, scale bars = 0.5 mm) and regions of interest in the cortex (overlay, scale bars = 50 μm) stained for NCC (magenta) and Parvalbumin (Parv, green) in (**A**) *Fam111a*^+*/*+^ and (**B**) *Fam111a*^−*/*−^ mice. (**C**) (i) Percentage of NCC-positive area and (ii) Parv-positive area of total kidney slice area and (iii) the ratio of the NCC-Parv positive area in *Fam111a*^+*/*+^ and *Fam111a*^−*/*−^ mice (n = 3 per group, all male mice).

The distal convoluted tubule (DCT) is important in determining the urine excretion of Mg^2+^ and Ca^2+^ by modulating transcellular reabsorption via TRPM6/7 Mg^2+^ channels and TRPV5 Ca^2+^ channels^25,26^. To study whether there are differences in the length of the distal convoluted tubule (DCT) between *Fam111a*^+*/*+^ and *Fam111a*^*−/−*^ mice, immunohistochemical staining was performed on kidney sections (Fig. 3A,B). The NCC-positive area per kidney slice area, which is an indicator for the total DCT segment size, was unchanged in *Fam111a*^*−/−*^ mice compared to the *Fam111a*^+*/*+^ group (Fig. 3C). The size of early DCT (DCT1) segments, based on parvalbumin-positive areas, was also comparable between *Fam111a*^*−/−*^ and *Fam111a*^+*/*+^ mice (Fig. 3C). The ratio of the DCT1:DCT2 cells was also not statistically different between *Fam111a*^+*/*+^ and *Fam111a*^*−/−*^ animals (Fig. 3C).

### Expression of transporter genes in the proximal duodenum and distal colon of Fam111a mice

**Figure 4:**
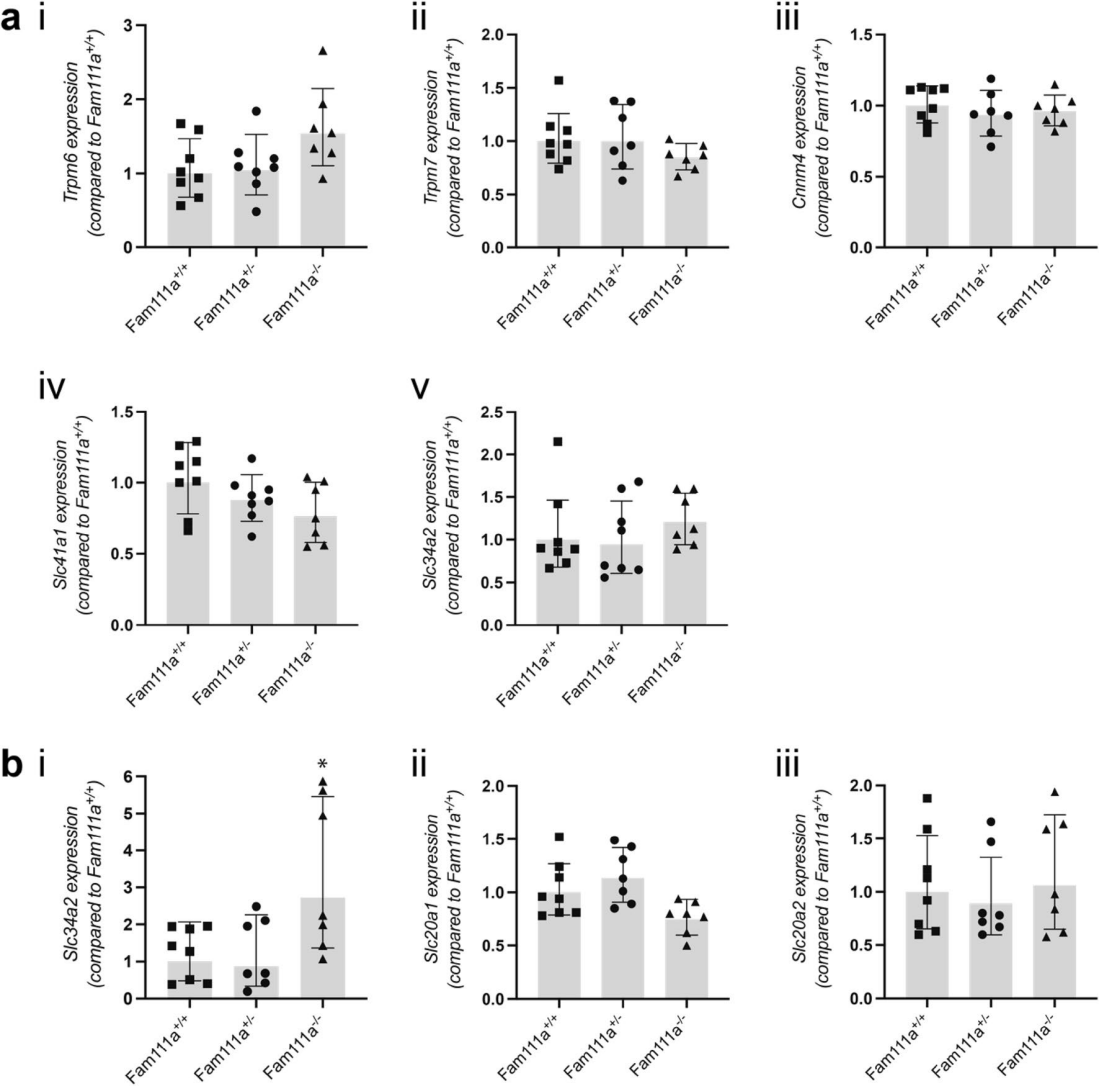
Expression of intestinal Mg^2+^ and PO_4_^3−^ transporter genes of *Fam111a*^−*/*−^ mice. (**A**) mRNA expression levels of (i) *Trpm6*, (ii) *Trpm7*, (iii) *Cnnm4*, (iv) *Slc41a1* and v) *Slc34a2* in the colon of *Fam111a*^+*/*+^*, Fam111a*^+*/*−^* and Fam111a*^−*/*−^ mice are unaltered. (**B**) Duodenal mRNA expression of the phosphate transporter (i) *Slc34a2* is significantly upregulated in *Fam111a*^−*/*−^ mice, whereas expression of (ii) *Slc20a1* and (iii) *Slc20a2* is unchanged. n = 8 (4 males, 4 females) for *Fam111a*^+/+^ and n = 7 (3 males, 4 females) for *Fam111a*^−/−^ mice. For colon *Trpm6*, *Slc41a1* and *Slc34a2* n = 8 for *Fam111a*^+*/*−^ (4 males, 4 females). For colon *Trpm7* and *Cnnm4* n = 7 (3 males, 4 females) for *Fam111a*^+*/*−^ mice, and for duodenal *Slc34a2*, *Slc20a1* and *Slc20a2* n = 7 (4 males, 3 females) for *Fam111a*^+*/*−^ mice. Results were normalised to *Gapdh* expression (reference gene). Data are presented as mean ± SD. Significance was determined using one-way ANOVA followed by Tukey’s post-hoc test. *p < 0.05 compared to *Fam111a*^+/+^ mice.

Electrolyte levels are regulated by changes in intestinal absorption. For this reason, expression of genes involved in electrolyte absorption was measured to identify differences between *Fam111a*^+*/*+^*, Fam111a*^+*/−*^ and *Fam111a*^*−/−*^ mice. The expression levels of *Trpm6*, *Trpm7*, *Cnnm4*, *Slc41a1* and *Slc34a2* in the distal part of the colon of *Fam111a*^+*/−*^ and *Fam111a*^*−/−*^ mice were similar to those in the *Fam111a*^+*/*+^ group (Fig. 4A). While the expression levels of phosphate transporters *Slc20a1* and *Slc20a2* were unchanged, *Slc34a2* expression was significantly upregulated in the *Fam111a*^*−/−*^ mice compared to the other groups (*p* < 0.05) (Fig. 4B).

### Fam111a^−/−^ mice exhibit a normal bone phenotype

**Figure 5:**
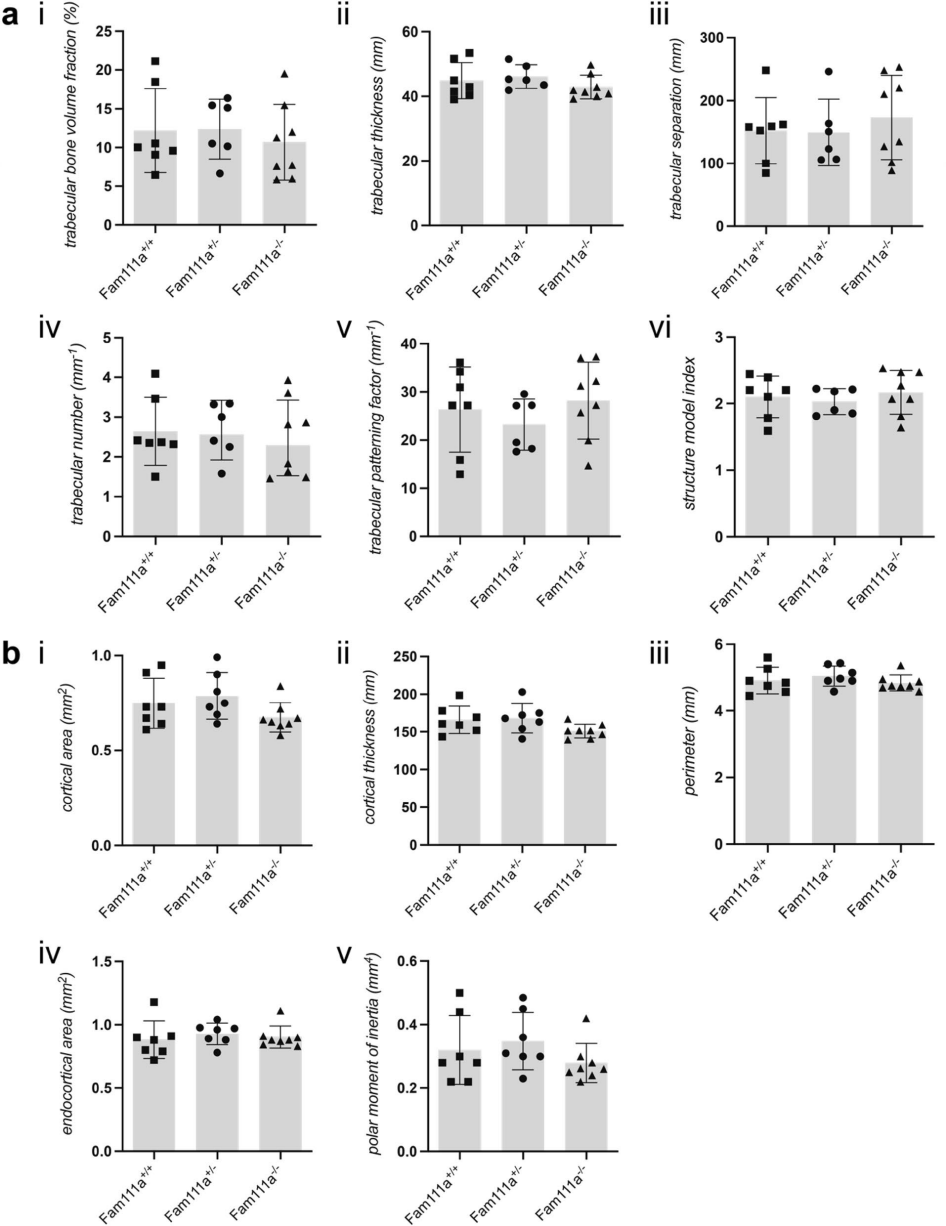
Microstructural properties of the femur from *Fam111a*^−*/*−^ mice are unaffected. (**A**) Trabecular and (**B**) cortical microarchitectural measurements in *Fam111a*^+*/*+^, *Fam111a*^+*/*−^ and *Fam111a*^−*/*−^ mice 8–10 weeks old. n = 7 (3 males, 4 females) for Fam111a^+/+^ and Fam111a^+/−^ and n = 8 (4 males, 4 females) for Fam111a^−/−^ mice. Data are presented as mean ± SD. Significance was determined using one-way ANOVA.

KCS2 patients have a particular bone phenotype including cortical thickening and medullary stenosis of the long bones. To investigate a potential role of FAM111A in skeletal development, micro-CT was used to characterise structural remodeling of femoral bone in *Fam111a* mice. Figure 5A shows trabecular parameters of *Fam111a*^+*/*+^, *Fam111a*^+*/−*^ and *Fam111a*^*−/−*^ mice. *Fam111a* deficiency did not affect trabecular bone volume fraction, trabecular thickness, trabecular separation, trabecular number and patterning factor of trabecular region (connectivity of trabeculae), or the structure model index (plate- *versus* rodlike appearance of trabecular bone).

The bone cortical indices were also monitored for structural changes (Fig. 5B). Cortical area, thickness, perimeter and the endocortical bone marrow area were not different in the *Fam111a*^*−/−*^ mice. The polar moment of inertia (a proxy for bone strength) was also the same among the groups. Taken together, in contrast to patient findings where increased cortical thickening was described, there are no observable alterations in the bone microarchitecture upon deletion of *Fam111a*.

### PTH levels and parathyroid tissue histology are unaltered in Fam111a^−/−^ mice

**Figure 6:**
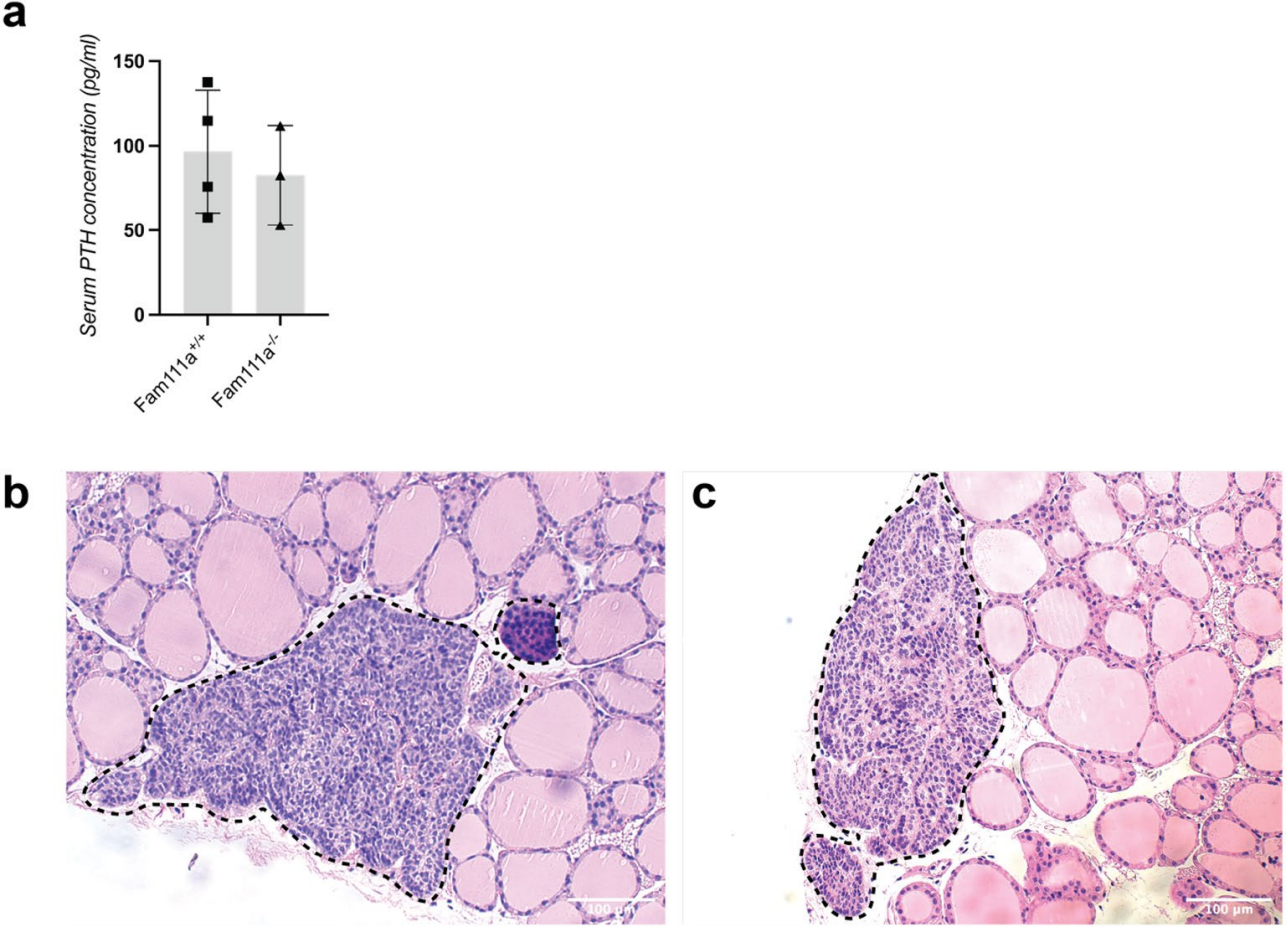
Parathyroid hormone levels and parathyroid histology in *Fam111a*^−*/*−^ mice. (**A**) Parathyroid hormone levels in serum of *Fam111a*^+*/*+^ and *Fam111a*^−*/*−^ mice. n = 4 for *Fam111a*^+*/*+^ and n = 3 for *Fam111a*^−*/*−^ mice (all male mice). Data are presented as mean ± SD. Significance was determined using an unpaired t-test. (**B**) H&E staining of thyroid tissue showing normal parathyroid tissue in *Fam111a*^+*/*+^ and (**C**) *Fam111a*^−*/*−^ mice. Parathyroid tissue is encircled by a dotted line.

While hypoparathyroidism is a common feature of KCS2, the PTH concentration in the serum of *Fam111a*^*−/−*^ animals was similar to that in the *Fam111a*^+*/*+^ group (Fig. 6A). In addition, we performed H&E staining on thyroid sections of *Fam111a*^+*/*+^ and *Fam111a*^*−/−*^ mice. This demonstrated that *Fam111a*^*−/−*^ mice had parathyroid glands of normal cellular constitution, consisting primarily of chief cells (Fig. 6B, C). We did not detect any damage such as cysts or immune cell infiltration to the parathyroid glands in *Fam111a*^*−/−*^ mice.

## Discussion

In this study, we investigated whether FAM111A plays a role in growth and electrolyte homeostasis to help explain the KCS2 patient phenotype. For this, we used a knockout mouse model. *Fam111a*^*−/−*^ mice showed normal body weight, bone morphology, electrolyte homeostasis and PTH levels. Based on our results, we conclude that FAM111A is not essential in regulating growth and electrolyte homeostasis in mice on a standard diet.

Expression analysis demonstrated that duodenal *Slc34a2* transcripts were significantly upregulated in *Fam111a*^*−/−*^ mice, but the contribution to total phosphate absorption of this increased expression is unclear as *Slc34a2* dependent phosphate absorption in mice mainly occurs in the ileum^30^. RNAseq of the kidney did not identify additional ion channels and transporters differentially regulated in *Fam111a*^*−/−*^ mice. While there were no changes in the mRNA expression of ion channels and transporters in this study, our data does not exclude the regulation of channel activity at the level of protein expression and/or post-translational regulation. The expression of *Glyat* and *Gm4952* were significantly downregulated. However, these genes are located in the vicinity of *Fam111a* on the genome. It is possible that there are enhancers present within the *Fam111a* gene that affect the expression of these genes. *Mid-1* and *Slc47a1* were upregulated, however, this change is of unknown significance to the KCS2 phenotype. A recent study using small sample single cell RNA sequencing (scRNAseq) of mouse kidney tissues identified a cluster of early DCT-like cells with high enrichment of cell cycle and replication factors suggesting a proliferative potential in these DCT cells^31^. However, total DCT area as well as total early DCT area was similar in the *Fam111a*^+*/*+^ and *Fam111a*^*−/−*^ mice.

Several explanations can contribute to the absence of the patient phenotype in *Fam111a*^*−/−*^ mice, including: (1) the nature of FAM111A mutations (loss of function vs gain of function), (2) compensatory mechanisms for *Fam111a* deletion, (3) differences between mouse and man. We will elaborate on these points in the following sections.

The exact consequences of the nature of the *FAM111A* patient mutations on the protein function are unclear: specifically, whether it leads to a gain or loss of protein function. Indeed, the presence of mutations in the FAM111A catalytic site in some KCS2 patients are in line with a loss of function effect^8,32^. However, recent in vitro studies have pointed towards a gain of function effect on the protease activity of FAM111A^18,20^. On the other hand, because the FAM111A protease activity can result in autocleavage, it has been also hypothesized that activating mutations in FAM111A eventually will result in loss of function by enhanced autocleavage^18^. The present study seems to support the viewpoint that a gain of function rather than a loss of function of FAM111A is responsible for the observed pathology, as we did not observe an electrolyte phenotype in the knockout mice. Mouse models of electrolyte disorders due to hyperactivating mutations such as autosomal dominant hypocalcemia (ADH) and familial hyperkalemic hypertension are generated by knock in of the relevant mutation in mice^33–37^. Similarly, transgenic knock in mouse models expressing the FAM111A patient mutations may better reflect the phenotype seen in patients with KCS2.

Based on our study, we cannot exclude that the function of FAM111A is rescued by compensatory mechanisms. FAM111A has been recently shown to function as a DNA–protein crosslinking (DPC) protease^18^. Spartan (SPRTN), the most well characterised DPC protease, possesses some overlapping functions with FAM111A in preventing replication fork obstacles by Top1-cleavage complexes (Top1cc) and formaldehyde^18,38^. Loss of function mutations in spartan result in Ruijs-Aalfs syndrome (RJALS) with features such as premature aging, cataracts, stunted growth, skeletal deformities and liver cancer^39^. However, unlike *Fam111a*^*−/−*^ mice, complete deletion of spartan in mouse models is embryonically lethal while hypomorphic mice develop symptoms of RJALS^40^. It is thus possible that unidentified proteases may compensate for the loss of FAM111A in mice.

Electrolyte imbalance is a prominent feature of Kenny-Caffey syndrome 2 and knockout mouse models have been employed extensively in elucidating the transport function of genes that encode transport proteins^41–44^. Also, knockout mouse models have been useful in identifying electrolyte related function of genes that do not encode for transporters (indirect regulation). For example, the roles of Adenylate cyclase 3 (*Adcy3*) and calcineurin in regulating Mg^2+^ handling were described using knockout mouse models^45,46^. However, not all knockout mouse models recapitulate the phenotype associated with the human disease. For example, *Fxyd2* knockout mice have a normal serum Mg^2+^ concentration, whereas loss-of-function mutations in *FXYD2* are responsible for isolated dominant hypomagnesemia in patients^47,48^. In addition, *Trpm6* knockout mice did not display hypomagnesaemia as is the case in the disease hypomagnesaemia with secondary hypocalcaemia (HSH) caused by mutations in *Trpm6*. Similar observations have been made in mice deficient in^49^ *Cnnm2*, suggesting that magnesium homeostasis in mice may be differently regulated compared to man. Mice are also commonly used to study parathyroid function, but Günther et al*.* found that glial cells missing homolog 2 (*Gcm2*) deficient mice and parathyroidectomised mice had normal serum PTH levels, as the thymus provided a backup mechanism of PTH secretion^50^. Indeed, mice harbouring a point mutation in *Tbce* had no parathyroid glands, but normal PTH levels^51^. Therefore, mice may not be a suitable model for studying the role of FAM111A in electrolyte handling.

FAM111A has been described as a DNA replication stress response protein in in vitro studies by preventing replication fork stalling caused by poly-ADP-ribose polymerase 1 (PARP1) accumulation as well as by responding to viral stress, preventing viral DNA replication^18,22,23^. Deletion of FAM111A in HAP1 cells did not affect cell viability and proliferation unless they were treated with DNA-damage agents^18^. It would be interesting to see how *Fam111a*^*−/−*^ mice would respond to stress conditions where FAM111A is known to play a role such as response to viral stress (LT-virus and orthopoxvirus) or DNA replication stress induced by PARP1 and/or topoisomerase 1 (TOP1) inhibitors. In addition, dietary restriction of electrolytes could be a trigger to develop electrolyte related symptoms. An example was described in serum- and glucocorticoid-regulated kinase 1 (SGK1) knockout mice which displayed subtle changes in electrolyte regulation compared to control mice in normal conditions and challenging them with dietary NaCl restriction impaired renal sodium handling^52^. Finally, DCT cells are known to structurally adapt to different stimuli, such as furosemide and low sodium or potassium diets^53–55^. Investigation of how these mice will respond to these stimuli may explain a potential role of *Fam111a* in DCT replication and remodelling.

In conclusion, our data show that *Fam111a*^*−/−*^ mice display normal electrolyte homeostasis, PTH levels, growth, and skeletal development. Expression of relevant electrolyte transporters was not altered in the kidneys and intestines. Hence, knockout of FAM111A does not affect renal electrolyte handling in mice on a standard diet.

## Methods

### Generation of *Fam111a*^−/−^ mice

The C57BL/6 N *Fam111a* mouse strains used for this research project were created from embryonic stem (ES) cell clone 19174A-G2, 19174A-A1 generated by Regeneron Pharmaceuticals, Inc. This ES cell clone was subsequently used by the KOMP Repository (https://www.komp.org/) and the Mouse Biology Program (www.mousebiology.org) at the University of California Davis to produce chimera mice. Methods used to create the Velocigene-targeted alleles have been reported previously^56^. The non-conditional allele Fam111a^tm1(KOMP)Vlcg^ was generated by insertion of ZEN-UB1 cassette targeting chromosome 19 in ES cells, which results in the deletion of 4,553 nucleotides (12,584,048–12,588,600; GRCm38.p6) corresponding to the entire protein coding region of *Fam111a*. ES cell clones were selected based on PCR genotyping and then blastocysts were microinjected into C57BL/6 N background mice. Balb/c donors were used for microinjection to produce the chimera. Sperm rederivation was performed to produce *Fam111a*^+*/−*^ animals. *Fam111a*^−/−^ and *Fam111a*^+*/*+^ mice were generated by breeding *Fam111a*^+*/−*^ mice.

The following primer pair was used for genotyping the wild type (*Fam111a*^+*/*+^) allele: forward primer (5′-AGCCAAAATGAGCTGTAAGAAGC-3′) and reverse primer (5′-GTGTCATTGTCCCATTCAAC-3′). The following primers were used for genotyping the presence of the ZEN-UB1 cassette: forward primer (5′-TCATTCTCAGTATTGTTTTGCC-3′) and reverse primer (5′-TGAACTTTCTCACATCGTAGC-3′).

### Ethics and approval

Animal studies were carried out at the Radboud Institute for Molecular Life Sciences, Nijmegen, The Netherlands. All methods and protocols performed in this study were approved by the local Ethics committee of the Radboud University Nijmegen (RU DEC 2015-0112-006) and the national Ethics committee of the Dutch Central Commission for Animal Experiments (AVD103002016382). This study was performed in compliance with the ARRIVE guidelines. The methods were in accordance with relevant guidelines/regulations, as follows. *Fam111a*^+*/*+^ mice were used as controls and individual mice were considered experimental units. Sample size was calculated as n = 7 per group based on the outcome measurement of serum magnesium, using a Java Applet^57^. We did not make use of any exclusion criteria and no animals were excluded, but one heterozygous animal died before the study. Randomisation was applied in housing and sample collection. Blinding was performed by giving all animals a unique number, so that the genotypes were not known to the researchers. All genotypes were randomly divided over the cages using the website https://www.random.org/sequences/. Sampling was done per cage. Researchers were blinded to the genotype until the first analyses of results. Mice were housed with maximally 6 animals in standard cages (mouse Eurostandard type IIL) and during the experiments they were individually housed for 48 h in metabolic cages (24 h of acclimatisation, 24 h for actual experiments), after which they were placed back into their own cages. Cage enrichment (bedding, nesting material and igloo) was used in the standard cages, but not the metabolic cages. Specific humane endpoints were defined as follows: (1) The animal has visible signs of discomfort, such as weight loss (more than 15% of the initial weight), hunchback, bad fur and/or bad movement pattern, (2) The animal loses more than 20% weight during housing in a metabolic cage, (3) Specific for the low serum magnesium value: the animal has visible muscle cramps, the animal shows lack of movement, the animal shows a severe abnormal walking pattern (wobbling), the animal has severe exorotation of the heel (indication of muscle weakness) or the animal is not able to hold on to the cage (indication of muscle weakness). These endpoints were monitored daily through weighing and visual monitoring.

### Metabolic cages and tissue sampling

Mice (8–10 weeks old) were placed in metabolic cages and left to acclimatise for 24 h. Mice were kept on a normal diet, containing 1% calcium (w/w), 0.22% magnesium (w/w), 0.7% phosphate (w/w) and 1000 IU/kg vitamin D_3_. During the next 24 h, the mice were weighed and urine and faeces were collected. In addition, water and food consumption were measured. The following day, the mice were anaesthetised by 4% (v/v) isoflurane inhalation, serum/blood was collected and the mice were sacrificed. Kidney, thyroid glands, duodenum and colon were collected and snap frozen in liquid nitrogen and stored at -80ºC until further analysis.

### Serum electrolyte and PTH concentrations

Serum and urine PO_4_^3−^, Na^+^ and K^+^ were determined by the clinical laboratory of the Radboud University Medical Center using an automated system (Abbott Diagnostics, Hoofddorp, The Netherlands). Mg^2+^ concentrations were determined by xylidil blue colorimetric assay kit (Roche/Hitachi, Tokyo, Japan) and read at a wavelength of 600 nm on a BioRad plate reader (BioRad, California, USA). Serum and urinary Ca^2+^ concentrations were measured using a chromogen-based colorimetric assay and read at a wavelength of 570 nm (Sigma Aldrich, Zwijndrecht, The Netherlands). The values obtained were calibrated using a Precinorm standard solution (Precinorm U, Roche, Switzerland). Serum albumin was measured using the BCG albumin assay kit (Sigma-Aldrich, Saint Louis, USA) according to the manufacturer’s recommendation and read at a wavelength of 620 nm on a BioRad plate reader (BioRad, California, USA). PTH serum concentrations were measured using the mouse PTH 1-84 ELISA kit (cat. No 60-2305, Quidel, CA, USA) according to the manufacturer’s instructions.

### Histology (haematoxylin & eosin)

Tissues were fixed for at least 24 h in 4% (v/v) formalin. Samples were dehydrated in ethanol, embedded in paraffin and cut into sections of 5 μm. The sections were deparaffinised in xylene for 10 min and then rehydrated in graded concentrations of ethanol (100%, 95%, 75%, 50% v/v). The sections were incubated 5 min in haematoxylin (Merck, Amsterdam, Netherlands) and differentiated in tap water after which the sections were stained with eosin (Merck) and dehydrated in in graded concentrations of ethanol (50%, 70%, 96%, 100% (v/v)). Slides were cleared in xylene and mounted using Pertex mounting medium (VWR, Amsterdam, The Netherlands). Tissue slices were imaged using a Zeiss Axiocam 503 color camera mounted onto a Zeiss Axio Imager M2.

### RT-qPCR

Total RNA was extracted from kidney and intestinal tissues using TRIzol reagent (Invitrogen, Bleiswijk, The Netherlands) according to the manufacturer’s protocol. The isolated RNA was treated with DNase (Promega, Madison, WI, USA) to prevent contamination from genomic DNA. RNA concentrations and quality were checked using the Nano-Drop 2000c Spectrophotometer (Thermo Fisher Scientific, Breda, Netherlands). To generate cDNA, 1.5 µg RNA was reverse transcribed using Moloney murine leukemia virus (M-MLV) reverse transcriptase (Invitrogen, Breda, Netherlands) at 37 °C for 1 h. Gene expression levels were quantified by SYBR-Green (BioRad, Hercules, CA, USA) on a CFX96 real-time PCR detection system (BioRad) and normalised for *Gapdh* expression. Relative mRNA expression was analysed using the Livak method (2^−ΔΔCT^) and annotated as fold change of expression compared to the *Fam111a*^+*/*+^ group. Primer sequences are provided in the Supplementary Table S1.

### RNAseq

Total RNA was extracted from kidney tissue using TRIzol reagent (Invitrogen) according to manufacturer’s protocol and described earlier. RNA samples from *Fam111a*^+*/*+^ (n = 4) and *Fam111a*^−/−^ (n = 3) male mice were sent for RNA sequencing. Quality control and RNAseq were performed by BaseClear B.V. (Leiden, The Netherlands). Counts of transcripts per sample were generated and quantified using Salmon and expressed as transcripts per million^58^. The reference transcriptome was downloaded from the BioMart repository at Ensembl (Cambridge, UK). The raw and normalized count data were further analysed using R (Vienna, Austria). Raw and processed data files are available in the Gene Expression Omnibus database (GSE196466).

### Micro-CT

Femurs were dissected and fixed in 4% (v/v) formalin for 24 h and subsequently kept in 70% (v/v) ethanol. Femurs were scanned using the Skyscan 1076 in vivo X-ray computed tomographer (Bruker microCT, Kontich, Belgium) with a voxel size of 8.88 μm and an exposure time of 2300 ms as described previously^49^. Briefly, X-ray power was set at 40 kV and tube current at 250 mA. Beam hardening (20%) was reduced using a 1 mm aluminum filter, ring-artefacts was set at 5 and an average of three photos (frame averaging) at each angle (0.8°) were taken to generate the final images. Cortical bone parameters were performed in the diaphyseal cortex which included a scan area of 0.45 mm in the femoral center. Trabecular bone measurements were obtained by scanning the distal metaphysis, a total scan area of 1.35 mm from the distal growth plate to the femoral center.

### Immunohistochemistry

Immunohistochemistry was performed as previously described^59^. Briefly, co-staining for parv and NCC were performed on 4 μm sections of formalin fixed and paraffin embedded kidney samples. Sections were incubated with blocking buffer (TSA fluorescence system, Perkin Elmer) to prevent unspecific antibody binding. Sections were incubated overnight at 4 °C in rabbit anti-parvalbumin (1:200, Swant, Bio Connect, Switzerland) and sheep anti-NCC (1:200, MRC) in blocking buffer. For detection, sections were incubated with Alexa Fluor-conjugated secondary antibodies (Thermo Scientific, Amsterdam, The Netherlands). Cell nuclei were stained with DAPI (1:20,000) for 10 min at room temperature. Images were taken with an AxioObserver camera and stitched with AxioVision software (Zeiss, Sliedrecht, The Netherlands). NCC or parv positive regions of interest were obtained by thresholding and total positive area per kidney slice was measured using the analyse particle function in Fiji^60^.

### Statistical analysis

Data are expressed as mean ± SD. Analysis was done using Graphpad Prism v8 (San Diego, CA, USA). For statistical analysis of three groups, the assumption of equal variances was tested using the Brown-Forsythe test and normality was tested using the Shapiro–Wilk test. For two groups, the assumption of equal variances was tested using an F test. If assumptions were not met, data was first logarithmithically transformed to achieve equal normality. Then, for three groups one-way ANOVA was used to test for differences in the data, followed by a Tukey’s multiple comparison test in case the null hypothesis was rejected. In the case of two groups, an unpaired t-test was used. Differences with a *p* value of < 0.05 were considered statistically significant. For the RNAseq, additionally, a false discovery rate (FDR) ≤ 0.05 was used to determine statistical significance.

## Supplementary Information

Supplementary Information.

## Data Availability

Raw data are available from the corresponding author, J.H.F.B., upon reasonable request. Raw and processed RNAseq data files are available in the Gene Expression Omnibus database (GSE196466).
