# Supplementary material for: FAM111A is dispensable for electrolyte homeostasis in mice
Source: Sci Rep. 2022 Jun 17;12:10211. doi: 10.1038/s41598-022-14054-8 (PMC9205974; doi:10.1038/s41598-022-14054-8)
Supplement: Supplementary file 1 — Supplementary Information. [file 41598_2022_14054_MOESM1_ESM.pdf]

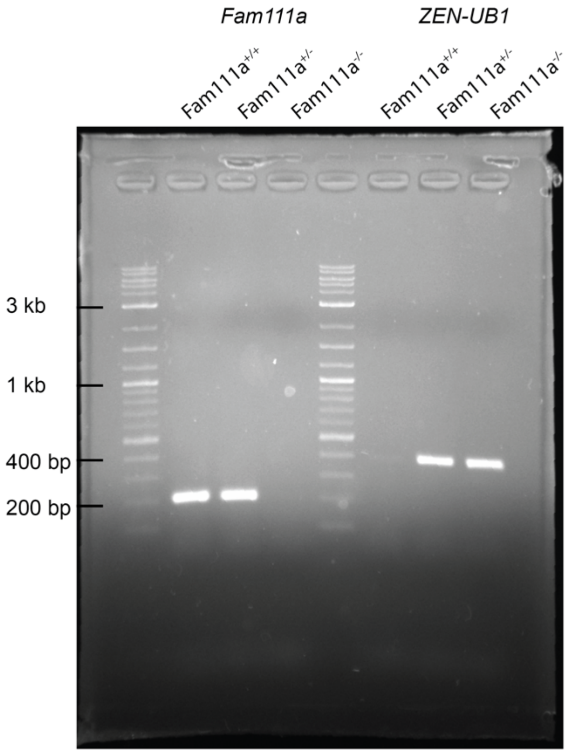

Supplementary Figure S1: Mouse genotyping

Full-length gel with PCR products showing the genotype of *Fam111a*$^{+/+}$, *Fam111a*$^{+/-}$ and *Fam111a*$^{-/-}$ mice at the expected sizes.

Supplementary Table S1. List of primers for qPCR

Target gene	Forward primer (5’-3’)	Reverse primer (5’-3’)
Gapdh	TAACATCAAATGGGGTGAGG	GGTTCACACCCATCACAAAC
Trpm6	AAAGCCATGCGAGTTATCAGC	CTTCACAATGAAAACCTGCCC
Trpm7	GGTTCCTCCTGTGGTGCCTT	CCCCATGTCGTCTCTGTCGT
Cnnm2	GTCTCGCACCTTTGTTGTCA	GTCGCTCCGACTGAGAGAAT
Cnnm4	TCTGGGCCAGTATGTCTCTG	CACAGCCATCGAAGGTAGG
Slc41a1	TCCCTGATGGCCACTTTAGC	GATCATACCCAGGACCAAGGAG
Slc34a1	TCAGGAAGAGGAGCAAAAGC	AAAGGAAAGCCAGCATCAGA
Slc34a2	CTATTCCGCCCTGGTTCTC	GAAAATGCAGAGCGTCTTCC
Slc34a3	GTGGTCAGCAGCTTTCTCAA	ACAGCACCACATTGTCCTTG
Slc20a1	TGTATTGTCGGTGCAACCAT	ATACCAGAAAGCAGCGGAGA
Slc20a2	CGGCGTGCTGTTCATACTAA	GCAGCATAAAACAGAGGCAGT
Pthr	TGACCAATGAGACTCGGGAAC	CAGTGCAGCCGCCTAAAATAG
Trpv5	TGCTCAACTTGTTCATTGCCAT	CCACAGGAAACGAGGCATTTT
Calbindin 28k	ATTTCCGGTGATAGCTCCAA	ATTTCCGGTGATAGCTCCAA
Klotho	GGTTGCCCACAACCTACTTT	TGGGAGCTTAAGGCGATAGA
Cyp24a1	GGAGTCCATGAGGCTTACCC	GGTAGCGTGTATTCACCCAGA
Cyp27b1	GTGTTGAGATTGTACCCTGTGG	TGGGGAATTACATAGTTTCCTACAC
Fgfr1	TAAGATCGGGCCAGACAACT	CGATAGAGTTACCCGCCAAG
Prl1	ATAAGACAAAAGCGGCGTGG	TGGAATCCTTGAAGCGGAGC
Prl2	CCAGCTTGCCAGCGTTTTTC	TGGCACGTATAACCCCAAGC
